# Pneumatosis Coli Associated with Pseudomembranous Colitis in a Patient following Colonic Surgery

**DOI:** 10.1155/2010/138369

**Published:** 2010-11-11

**Authors:** Jennifer Bailey, Eldon A. Shaffer

**Affiliations:** Division of Gastroenterology, Faculty of Medicine, University of Calgary, Teaching Research & Wellness Building, Room 6D48, 3280 Hospital Dr NW, Calgary, AB, Canada T2N4N1

## Abstract

Pneumatosis intestinalis is a rare disorder characterized by gas-filled cysts within the subserosal and/or submucosal regions of the intestinal wall. The source of this gas and its translocation across the mucosa is incompletely understood. Most (85%) cases are associated with medical conditions, ranging from psychiatric through respiratory disorders to gastrointestinal-related diseases; the remaining 15% lack any recognizable cause or association. In this case report, pneumatosis coli (affecting the colon) occurred in a patient following abdominal surgery and was associated with pseudomembranous colitis, which was *Clostridium difficile* toxin negative—presumably a false negative. Supportive care and appropriate antibacterial agents sufficed to alleviate symptoms and resolve the pneumatosis. Recognizing this uncommon but important association can avoid high financial and personal costs from unnecessary testing and invasive surgical explorations. Consideration should be given to pseudomembranous colitis as the basis for pneumatosis coli developing in patients who have received antibiotics, once gut ischemia has been ruled out.

## 1. Introduction

Pneumatosis intestinalis (PI) is characterized by multiple, thin-walled, gas-filled cysts within the subserosal and submucosal regions of the intestinal wall. Typically the location of pneumatosis is 46% in the colon, 27% small bowel, 5% stomach (usually termed gastric pneumatosis), and 7% involving both the small and large intestine [1]. Its pathogenesis is not completely understood. 15% of cases are primary or idiopathic, without identifiable cause or association. 85% of cases are associated with a wide variety of medical conditions and infections, suggesting a number of potential underlying pathogenic processes that may contribute to its development, that is, as a secondary event [2]. When limited to the colon, such collections of intramural gas are termed pneumatosis coli (PC). 

We report a case of PC in a patient following colonic surgery, which was diagnosed as pseudomembranous colitis and successfully treated for a toxin-negative *Clostridium difficile* infection.

## 2. Case Presentation

**Figure 1:**
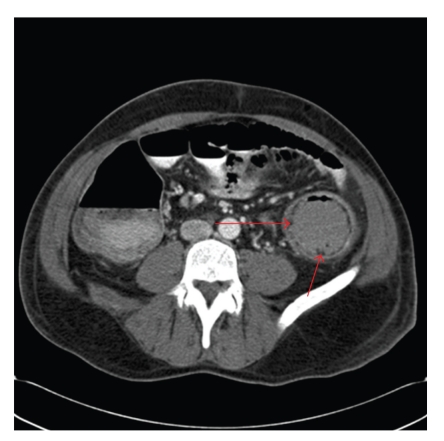
Abdominal computed tomography (CT) revealing submucosal air-fluid cysts. Arrows demarcate submucosal gas within the wall of the left colon, pneumatosis coli.

**Figure 2:**
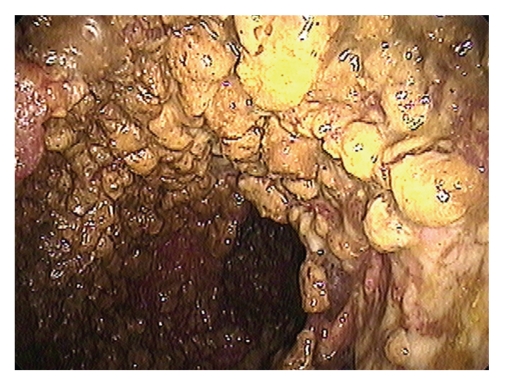
Pseudomembranes and pneumatosis coli. Endoscopic evaluation of the colonic mucosa reveals pseudomembranes. Cystic lesions are evident beneath the pseudomembranous layer.

A 56-year-old male with recently diagnosed rectal carcinoma (T1N0M0) underwent a low anterior resection and diversion ileostomy that was followed four weeks later by an uncomplicated loop ileostomy closure. Within a week of this reversal surgery, he was readmitted with nausea, vomiting, anorexia, and diffuse abdominal pain, along with 6–8 nonbloody, small volume, liquid bowel movements daily. The patient had not been taking proton pump inhibitors, but had received ceftazidime and gentamicin with his recent surgeries. He appeared unwell on admission with nonspecific abdominal tenderness upon palpation; bowel sounds were normal. His hemoglobin was 130 g/dL, platelet count 416 × 10^9^/L, and white blood cell count 6.7 × 10^9^/L. Electrolytes, including bicarbonate, and creatinine were also within normal limits. The initial stool collections, were negative for the *C. difficile* cytotoxin assay (*C. difficile* Tox A/B II (Techlab, Blacksburg, VA)), bacterial culture, and ova/parasites. Abdominal X-rays revealed distended loops of large bowel with mural edema. Subsequent abdominal computerized tomography (CT) demonstrated extensive pneumatosis coli (Figure 1) involving the descending colon; there was also thumb printing and dilated loops of bowel. The patient was initially placed on metronidazole 500 mg IV q8h for three days due to concern regarding the clinical suspicion for a *Clostridium difficile* associated diarrheal (CDAD) infection. The intravenous route was chosen because of the postsurgical ileus with abdominal distension, reduced bowel sounds, and inability to tolerate oral feeds. A mild metabolic alkalosis was present on blood gas but there was no evidence of acidosis that might have suggested a gut ischemia. Flexible sigmoidoscopy clinically confirmed the diagnosis, showing quite classical pseudomembranes in addition to features consistent with pneumatosis intestinalis (Figure 2). Biopsies revealed a focal active colitis with superficial ulceration, gland destruction, and areas in which exudates overlay the colonic mucosa, all supporting the diagnosis of pseudomembranous colitis. Two subsequent *C. difficile *toxin assays however proved negative. The intravenous metronidazole was then changed to oral vancomycin at 125 mg four times daily for four days. The patient improved over the subsequent week with a reduction in the frequency of bowel motions and a marked improvement in abdominal discomfort. Follow-up abdominal CT scan 8 days after the initial study revealed resolution of the pneumatosis coli. Discharged thereafter, he remained well without a recurrence of either entity; his last colonoscopy was normal 7 months following discharge.

## 3. Discussion

### 3.1. Epidemiology

Pneumatosis intestinalis, rather than being a disease *per se*, better relates to the underlying pathological process that varies widely: from being quite incidental without symptoms detected on screening colonoscopy to being associated with life-threatening conditions, such as necrotizing enterocolitis in infants or ischemic/infracted bowel in adults [3]. Though a rare event, pneumatosis has historically mandated surgical exploration and was associated with a high mortality rate (33%), mostly reported as small case series [1]. A more recent study that reviewed 25,000 CT scans done over a 7-year period identified a prevalence of PI in 0.37% of patients [1]. Approximately 50% of these patients however were successfully managed nonoperatively. As many cases of pneumatosis are incidental findings detected on diagnostic imaging, its true frequency must be underestimated.

### 3.2. Clinical Associations

PI in adults is typically divided into primary (15% of cases being idiopathic, lacking any identifiable association) and secondary (85%) forms with diverse clinical associations, including psychiatric disorders, respiratory conditions, dementia, inflammatory bowel disease, diverticular disease, redundant sigmoid colon, appendicitis, peptic ulcer disease, and within the past decade, bacterial infections like *C. difficile * [4–6]. PI affecting infants is life-threatening, occurring in the setting of necrotizing enterocolitis as well as PC that usually presents as a milder form of this entity [7].

### 3.3. Pathophysiology

These multiple, gas-filled cysts (actually pseudocysts: spaces without a distinctive epithelial membrane) develop in the intestinal submucosa and subserosa, presumably by air or gas translocating into the bowel wall [8, 9]. A mechanical theory raises the concept of mucosal disruption through direct trauma or increased intraluminal pressure, permitting intraluminal gas to dissect directly into the submucosa or track via mesenteric vessels into the subserosa [8]. Such mucosal disruption could result from an endoscopic procedure, a perforated ulcer, inflammatory bowel disease, necrotizing enterocolitis, intestinal ischemia, diverticulosis, and after intestinal anastamosis, all associated with the development of pneumatosis. Nevertheless, there has been no documented anatomic connection between the mucosa and the cysts. A pulmonic theory invokes rupture of a pulmonary bleb though increased intrathoracic pressures (e.g., coughing), allowing air to transect through the mediastinum into the retroperitoneum, the perivascular spaces and thence the subserosa. Unexplained are the paucity of reports showing mediastinal air on diagnostic imaging [10]. Furthermore, a bacterial theory suggests that luminal bacteria enhance gas production (e.g., hydrogen gas from fermented carbohydrates) and this endogenous gas then diffuses across the mucosa [8]. In animal models, introduction of gas-forming bacteria can induce PC [11–14], which resolves with subsequent antibiotic therapy [12, 13, 15]. This mechanism however would presumably only explain PC, as opposed to PI, because hydrogen-producing bacteria reside in the colon and not the small bowel.

### 3.4. Clinical Presentation

The clinical picture can be quite varied depending on the underlying etiology and if any complications have transpired relative to the pneumatosis. The spectrum of pneumatosis nevertheless does include abdominal sepsis and death, often associated with evidence of portal venous gas [1, 2, 16]. Most commonly, patients have diarrhea, mucus discharge, rectal bleeding, and constipation [6]. Complications associated with PC include pneumoperitoneum, volvulus, intestinal obstruction, perforation and intussusception. In infants, pneumatosis intestinalis that develops secondarily to necrotizing enterocolitis culminates in a high mortality rate.

### 3.5. Diagnosis

Investigations should start with plain abdominal X-rays that may reveal complications related to PC; these are present in 23% [1]. Such findings include radiolucent clusters, linear streaks, or small bubbles along the bowel wall. If a cyst ruptures, a pneumoperitoneum develops. Barium enema might demonstrate the gas-filled cysts in the wall of the colon, but is not routinely ordered. Instead, CT imaging typically follows the abdominal X-ray in clinical practice. CT accurately identifies the bubble-like collections of gas within the submucosal and subserosal layers of the colonic mucosa. Evidence of pneumatosis when combined with prominent portomesenteric venous gas represents ominous findings, suggesting transmural bowel infarction. These impressive radiologic features however are not absolute prognostic indicators as CT imaging detects pneumatosis at a relatively early stage that may only signify partial mural bowel ischemia [16]. Care must be taken to also differentiate bubble-like PI from pneumatosis linearis: bands of gas within the bowel wall that place here associated with loss of gut viability, prompting urgent surgery. Ultimately an endoscopic evaluation may become necessary to differentiate PC from familial adenomatous polyposis and colonic malignancies. Intestinal biopsies in PI characteristically reveal gas-filled spaces within the submucosa that on histopathology are lined with histiocytes and multinucleated giant cells when involving the subserosa.

### 3.6. Diagnosis of Clostridium Difficile Infection

Key in managing PC is to identify a correctable etiology. In the present case, suspicion of a *Clostridium difficile* infection (CDI) arose because watery diarrhea had developed in a recently hospitalized patient who had received antibacterial agents [17, 18]. (Watery diarrhea in this context has been defined as ≥3 stools per day that are sufficiently liquid to conform to the shape of its laboratory container [16, 17]). The diagnosis of pseudomembranous colitis however came from diagnostic imaging, the endoscopy showing pseudomembranes, and the characteristic histopathology findings. Our patient had *C. difficile *toxin assays that were negative on three separate occasions.

*C. difficile *is a gram positive, rod-shaped bacterium. CDI results from pathogenic strains that are capable of producing two structurally similar toxins: A and B. Both are important in producing *C. difficile *colitis: toxin A being considered enterotoxigenic whereas toxin B has potent cytotoxic effects [19]. Hence, the laboratory diagnosis comes in two formats: (1) Toxin assays for one or both toxins, or for the cytotoxic effect, and (2) Microorganism assays to test for a common antigen or perform an anaerobic stool culture for *C. difficile. * Commercially available enzyme immunoassays (EIAs) are capable of detecting both toxin A and toxin B, important as occasionally some strains produce only A or only B. [20]. Although EIAs are rapid, inexpensive, and yield high specificity (up to 99%), their rather modest sensitivity (32–73%) results in a high false negative rate [18]. Repeating the stool EIA, as was done here when the initial test was negative, may improve sensitivity but only slightly (~10%). Re-ordering such stool tests moreover ultimately reduces specificity to unacceptable levels [17, 18]. More sensitive polymer chain reaction (PCR) assays for the toxin genes, recent additions, provide high sensitivity in the order of 97%. Lastly, tissue culture to detect cell cytotoxicity is costly and impractical despite being a “gold” standard for diagnosing *C. difficile*. 

 The second category checks for the presence of the organism. Common antigen testing seeks to detect the enzyme, glutamate dehydrogenase (GDH), that *C. difficile *produces [21]. EIA assays for GDH carry a high sensitivity (85–95%) while ensuring a high negative predictive value [21]. The GDH assay (like the cumbersome anaerobic stool culture) unfortunately also detects non-toxigenic and hence clinically irrelevant isolates. A strategy suggested by the 2010 IDSA guidelines on *C. difficile* supports a two step method: EIA to detect GDH as the initial screen (which is most sensitive), followed by a cell cytotoxicity assay, toxigenic culture or PCR for the toxin gene as the confirmatory test for GDH-positive specimens only, hence eliminating false-positive GDH results [21]. Culturing for *C. difficile *may be sensitive (over 90%), but is not practical as a routine diagnostic test since the results require 48–72 hours to complete. Further, anaerobic stool culture does not differentiate between toxin-producing and non-toxigenic strains. Culture is best reserved for epidemiologic, infection control or antibacterial studies.

 As no test combination to date is 100% sensitive and specific, endoscopy is an important diagnostic tool. Morphological diagnosis, as was necessary in this difficult case, came from the endoscopic picture and biopsy. Nevertheless, pseudomembranes are only detected endoscopically in about 50% of CDAD cases, as confirmed by both stool culture and cytotoxin stool test positivity [22]. Conversely, the presence of pseudomembranes must be taken in the clinical context, being neither absolutely sensitive nor specific for *C. difficile *colitis; pseudomembranes can accompany ischemic colitis and inflammatory bowel disease.

Some endoscopic similarities exist between ischemic colitis and CDI-associated pseudomembranous colitis, further complicating such difficult acute abdominal diagnoses. In ischemic colitis, however, any pseudomembranes tend to be more diffuse and have transmural involvement, compared to *C. difficile *colitis. Further, the histological presence of atrophic microcrypts, lamina propria hemorrhage, full-thickness necrosis and hyalinized lamina propria are specific markers for ischemia [23]. 

The diagnosis of pseudomembranous colitis in the current case is secure from the endoscopic and morphological perspectives. The presence of pseudomembranous colitis is strongly suggestive of toxigenic *C. difficile* infection regardless of the results of toxin EIA despite the absence of backup tests, such as cytotoxicity, culture, or PCR. Therefore, one of two conclusions is possible. (1) This was a toxigenic infection with a false-negative EIA—most likely, or (2) this was an extremely rare case of pseudomembranous colitis due to a cause other than C. difficile.

### 3.7. Prognosis and Management

The course of pneumatosis is quite variable, largely determined by the underlying disease process, especially those leading to bowel infraction or perforation. Traditionally, a diagnosis of PI was associated with a high mortality rate and mandated aggressive surgical intervention. In fact, the clinical course is generally quite benign: most (~50%) cases of PI spontaneously resolve, particularly when idiopathic. Regardless of PI location or intervention, there remains a significant mortality at 20%–40%, emphasizing the need to differentiate between sinister gut ischemia and rather benign PI [1]. Hence, interventions for imaging-diagnosed PI generally may be categorized as (a) operative (about 1/3rd), (b) nonoperative (~1/2), and (c) futile (~15%), favoring non-surgical management such as inserting a nasogastric tube supported by IV fluids.

The key decision therefore is whether to perform an emergency laparotomy for an acute intra-abdominal catastrophe or to manage the case conservatively for rather benign PI. A quite reasonable guide to identify gut infarction and the need for surgical intervention is a clinical setting that suggests an acute abdomen, laboratory evidence of a metabolic acidosis (particularly lactic acidosis) and elevated amylase, and diagnostic imaging showing linear pneumatosis and/or portal venous gas [24]. 

Specific treatment is best directed at the underlying disease process. For those with symptoms, management strategies directed at the pneumatosis itself have used (1) Oxygen therapy (either by inhalation or as hyperbaric therapy, the latter to reduce toxicity) for several days to impede anaerobic bacteria that produce the hydrogen and other gases in these cysts, and to replace these gases [11]; (2) antibacterial agents (like metronidazole) 7 days for up to 2-3 months to decrease bacterial gas production [12, 25]; (3) elemental diet for 2 weeks, seeking to alter the colonic microflora [26]. Colonic resection is occasionally necessary for complications like bowel obstruction. For the majority without symptoms, an expectant approach is all that is necessary because the intramural gas should resolve with time.

## Figures and Tables

**Figure 3:**
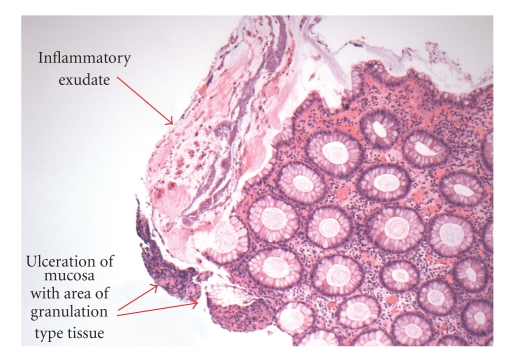
Colonic biopsy (10x) showing changes with exudate and ulceration (arrows) that are typical of the pseudomembrane formation seen in pseudomembranous colitis.
